# Success4life Youth Empowerment for Promoting Well-being and Boosting Mental Health: Protocol for an Experimental Study

**DOI:** 10.2196/38463

**Published:** 2022-09-14

**Authors:** Sajita Setia, Daniel Furtner, Mounir Bendahmane, Michelle Tichy

**Affiliations:** 1 Executive office Transform Medical Communications Wanganui New Zealand; 2 Transforming Life LLC Wilmington, DE United States; 3 Rollins College Winter Park United States; 4 University of Central Florida Orlando United States

**Keywords:** learned optimism, mind power tool, mental health, success4life, positive psychology-based interventions, well-being, youth empowerment

## Abstract

**Background:**

There is an increasingly alarming worsening of mental health among the youth. There remain significant unmet needs for developing innovative, evidence-based technology–enhanced, positive psychology interventions (PPIs) all-inclusive in targeting psychological distress and risk factors related to high-risk behavior commonly encountered in adolescents.

**Objective:**

We aim to assess the effectiveness of a hybrid (incorporating both synchronous and asynchronous learning) and holistic (targeting social and emotional learning and tackling risk factors unique for this age group) PPI, “success4life youth empowerment,” in improving well-being in the youth.

**Methods:**

Students’ well-being will be assessed by the 5-item World Health Organization Well-Being Index, and hope will be assessed by the 6-item Children’s Hope Scale at week 0, week 8, and week 10, month 6, and month 12. Any improvement in well-being and hope will be measured, estimating the difference in postintervention (week 8 and week 10) and preintervention (week 0) scores by determining the *P* value and effect size using appropriate statistical tests.

**Results:**

This study includes 2 phases: pilot phase 1, delivered by the creators of the succcess4life youth empowerment modules and platform, and phase 2, which will consist of the estimation of scalability through the recruitment of trainers. We hope to start student recruitment by 2022 and aim to complete the results for phase 1 pilot testing by 2023.

**Conclusions:**

We anticipate that a primarily web-based, 10-week holistic PPI can support improvement in the mental wellness of the youth and has the potential for effective scalability.

**International Registered Report Identifier (IRRID):**

PRR1-10.2196/38463

## Introduction

Globally, suicide is the second leading cause of mortality in adolescents and young adults [1]. A cross-national pooled estimate of the suicide mortality rate across all ages, sexes, and countries was found to be 3.77 per 100,000 people in 2019 [2]; however, underestimation and underreporting of youth suicide impact the accuracy of suicide epidemiology [3]. 

A bidirectional relationship exists between mental disorders and excessive social media use, substance use, and drug dependence, leading to a vicious cycle impacting youth across all communications and countries [4-7]. It has been postulated that even half of the mental and substance use disorders burden cannot be prevented with current treatments, regardless of the amount of funding available [8]. Data from across the world indicate a further increase in mental health–related emergency visits and reported suicidal ideation and behavior in the youth after the COVID-19 pandemic [1,9-12], despite a decline in screening for suicidal ideation during the pandemic [13]. A study based on a purposive sampling of Google News has revealed that web-based schooling, overwhelming academic distress, and TikTok addiction–related psychological distress are the leading stressors for suicide causalities in the pandemic [9].

The recent action plan on public mental health interventions by the World Psychiatric Association has laid crucial importance on preventing mental disorders and collaboration in delivering these interventions through educational organizations [14]. Primary prevention interventions prevent mental disorders from arising through the promotion of protective factors for mental well-being. Furthermore, as the COVID-19 pandemic fits the definition of a mental health disaster [15], we need innovative and cost-effective universal solutions for enhancing mental wellness that can be exercised at the population level.

Interventions for preventing mental and substance use disorders (MSUDs) during adolescence and early adulthood can be beneficial for promoting community mental well-being as most mental disorders start in this age group [8]. Positive psychology interventions (PPIs) lead to an increase in positive emotions, promote positive functioning, and lead to a reduction in stress and anxiety levels [16,17]. Any positive change in community mental health also leads to overall health, social, and economic benefits through improved productivity, resilience, educational attainment, and reduced risk-taking behavior [18]. PPIs promote well-being among individuals; however, community-level programs based on positive psychology vary greatly in their overall effectiveness [19,20]. Furthermore, the PPIs are typically brief and focus on a single element of positive psychology; for example, gratitude mindfulness, character strengths, or social and emotional skills [21-23]. While some PPIs, such as KidsMatter [24], which operate through improving social and emotional skills, have been particularly helpful for primary schools and early childhood services in Australia, there appears to be a lack of holistic PPIs that not only improve social and emotional skills but also tackle the issues related to social media and drug use for adolescents and young adults. The Hummingbird project [18] in the United Kingdom is an effective universal program for improving adolescent social and emotional skills. Nonetheless, it lacks broader coverage for preventing hazards through social media and awareness of the dangers of drug use. It also lacks a hybrid model incorporating technology-enhanced delivery.

Enthusiasm for technology in mental health–related services has evolved because of its promise to increase the reach of the scalability of services [25,26]. Although technology is regarded as a “new frontier in mental health” [25,27-29], both synchronous and asynchronous learning have pros and cons [30]. Synchronous learning refers to an instructional method wherein the learners interact with the instructor (through the internet or in person) in real time, giving a classroom-like feel. In contrast, asynchronous learning involves interactive learning where the learner and the instructor do not meet at the same time [31]. While synchronous learning permits personal real-time engagement that is more engaging and clarifies misunderstandings and doubts between learners and trainers, the coaching is available for a brief, limited period. On the contrary, asynchronous learning provides more time, a relaxed schedule, and flexibility in accessing educational material.

Technology-enhanced hybrid interventions continue to hold promise for increasing engagement in and enhancing outcomes of evidence-based approaches [25]. We have developed a hybrid PPI “success4life youth empowerment” that adopts unique benefits from real-time synchronous and asynchronous learning and overcomes the individual limitations of each coaching mode. This PPI is also holistic in the spectrum of coverage of elements of positive psychology and targets unique risk factors related to high-risk behavior commonly encountered in this age group. We aim to assess the effectiveness of this PPI in improving well-being and levels of hope in the youth.

## Methods

### Intervention Course

Earlier, we formulated an evidence-based course curriculum (success4life) for the prevention of MSUDs in the youth [8]. The ultimate objective of the curriculum is the universal prevention (applicable to everyone in the population) of MSUDs in the youth. We have now pioneered the modules, systems, and processes for effectively delivering the success4life program for adolescents and pipeline for emerging adults.

The entire curriculum is designed to provide hands-on coaching on adaptive coping for relieving stress, self-compassion, goal attainment, learned optimism and resilience, and self-protection, including awareness of the dangers of substance abuse and vigilance on social media. Coaching methodology includes hands-on experience in positive psychology with inquiry and project-based, student-directed learning through reflection within real-world experiences and problem-solving. The modules are created using evidence-based, scientific information published in peer-reviewed medical and scientific journals and esteemed books. The bibliography is included after each chapter of the modules. The content incorporated in each module and learners' journal template is protected by intellectual property, granted to Transforming Life LLC, United States (United States copyright office, registration number TXu 2-327-132; approval date July 27, 2022).

Our learning model follows blended digital learning, a combination of synchronous live real-time training and asynchronous e-learning via a learning management system (LMS), providing students expanded access to each week’s coaching. Live, real-time training allows sufficient contact with the instructors for effective interactive coaching and learning. The total duration for each weekly session of live training is between 60 and 90 minutes. Live, real-time training can be organized in digital (using videoconferencing technologies) and face-to-face settings. The ideal learner cohort size for group training varies depending on the setting (smaller learner cohort sizes in digital learning environments). During each week’s training, a structured approach is followed for simultaneous project completion. The entire training module is split into 2-4 chapters with journal exercises (assignments) throughout all the chapters. As relevant for some modules, separate project-based learning (PBL) is adopted to enable learners’ practical investigations, independence, and reflection within real-world practices. For submission of each week’s assignment and course projects, an effective LMS that hosts video modules for asynchronous refresher education and tracks data related to learner’s assignment submission, assignment evaluation, and workshop completion certification is used. Students’ privacy from the rest of the peers in the group for inquiry-based learning (IBL) through journal completion and submission is critical, and the activity completion is autonomous and independent for each learner. The learners’ data privacy will be ensured per local jurisdiction, and the management will collect users’ data only for the intended purposes.

The tactical curriculum based on the success4life program (currently marketed by Transforming Life LLC, United States) is designed to impart psychological and mental well-being through adaptive coping for relieving stress, self-compassion, goal attainment, learned optimism and resilience, self-protection, and refraining the vulnerable youth from high-risk behaviors, including addiction to devices, social media, and drug abuse. While other programs aim to enhance well-being in the youth, they neither follow the holistic success4life curriculum nor adopt an integrated framework encompassing the balance of synchronous learning, asynchronous learning, real-time IBL, and PBL. Further details on this concept can be understood using the animation available on YouTube [32].

### Modules of the Course

A broad overview of the theme, objectives and chapters is provided below. Module-specific details, including specific IBL and PBL exercises, are illustrated in Multimedia Appendix 1.

#### Week 1 Workshop: Understanding the Most Complex Yet Extremely Powerful Resource

The overarching objective of this workshop is to give students an awareness and appreciation of our innate potential, which can be harnessed by learning to control and shape our minds and thought processes. Three lessons are included: (1) understanding our mind, (2) the mind-body problem, and (3) the power of our mind.

#### Week 2 Workshop: Unlocking Your Potential

The overarching objective of this workshop is to provide an understanding of automatic, unconscious thought processes that are generated with little or no awareness and consciousness. The students also learn how to foster a good connection between the conscious and unconscious minds for success and happiness in life. Three lessons are included: (1) understanding our unconscious mind, (2) reprogramming our unconscious mind, and (3) how to apply knowledge in real-life situations.

#### Week 3 Workshop: Self-transformation

Given all the uncertainties at the current time, we may encounter feelings of hopelessness in our lives. However, each of us can “live an extraordinary life” irrespective of life’s challenges. In this workshop, the students learn why and how to expand our consciousness to achieve self-transformation with a deeper fulfillment and purpose. Three lessons are included: (1) what is self-transformation; (2) the relationship between self-transformation and happiness, abundance, and success; and (3) transforming the old self into a new empowered self.

#### Week 4 Workshop: Building a Sense of Self

The overarching objective of this course is to give students an awareness and understanding of how building self-esteem through self-compassion relates to positive outcomes in life and learn strategies to improve our self-esteem and self-confidence. Three lessons are included: (1) understanding self-image, self-esteem, and self-compassion; (2) building self-esteem and self-confidence through self-compassion; and (3) practicing self-love and self-care.

#### Week 5 Workshop: Protect Yourself

The overarching objective of this workshop is to give students an awareness and understanding of how our connections through friends, peers, and social media influence our thoughts, choices, and decisions in every way. The impact may be positive or negative, but we are often unaware of this dominance. In this module, students learn to exercise independence in their decisions without peer pressure and resist negative influences. Three lessons are included: (1) conscious connections and due diligence, (2) stay safe: substance abuse awareness, (3) it is okay to say no, and (4) staying vigilant on social media.

#### Week 6 Workshop: Prioritize, Energize, and Recharge Yourself

In this workshop, the students understand what it is like to practice reflection on their thinking with no or minimal judgment, allowing them to capture each moment with an awareness of change. They also learn to detach from nonbeneficial thoughts such as anger and judgment and let go of unwanted emotions. Three lessons are included: (1) building self-esteem, optimism, and resilience with mindfulness practices; (2) practicing the shield of mindfulness; and (3) meditation exercises.

#### Week 7 Workshop: Setting and Achieving Goals

In this workshop, the students understand the critical elements related to short- and long-term goals and build on setting personal goals for both the short and long terms. Three lessons are included: (1) discover your passion and purpose, (2) long-term goals, (3) short-term goals, and (4) what stops us from achieving our goals.

#### Week 8 Workshop: Tactics and Strategies to Achieve Goals in Life

The overarching objective of this workshop is to explain optimism and introduce students to a model to cope with life adversities, teach how to overcome negative thoughts, and convert adversities into opportunities with accurate choices and decisions. Four lessons are included: (1) definition and meanings of “optimism” and “resilience,” (2) the ABCDE (Adversity-Beliefs-Consequences-Disputation-Energization) model to cope with life adversities, (3) transforming your self-talk, and (4) how to turn adversities into opportunities with accurate choices and decisions?

#### Weeks 9 and 10

Weeks 9 and 10 include draft and final project presentations by students. Parents are invited to attend the last session. Course completion certifications are awarded for weeks 1-8, upon marking journal assignments completed in the LMS by the instructors. Another reward to the students who complete the entire program successfully includes eligibility into a peer mentor development program.

### Study End Points and the Road Map for Their Evaluation

**Figure 1:**
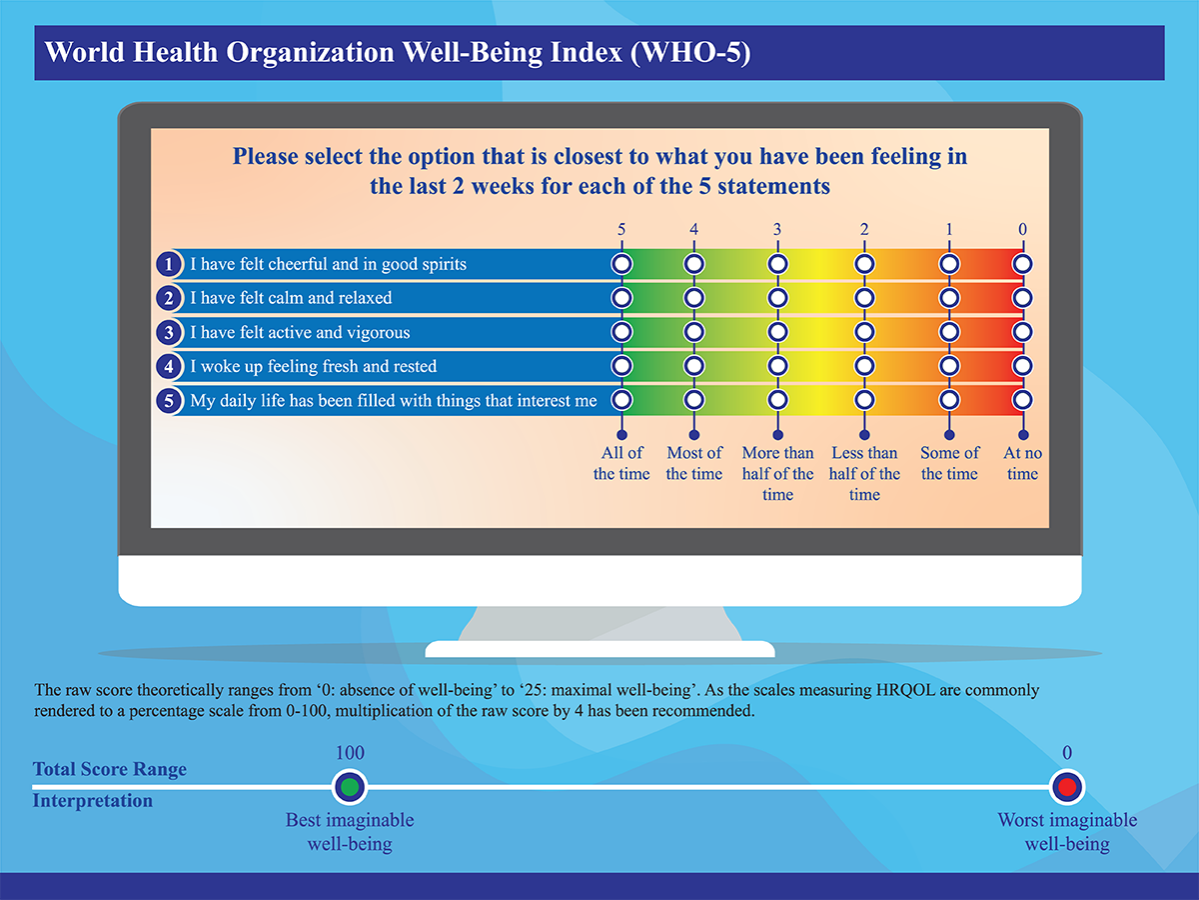
Graphical representation for the WHO-5 index. HRQOL: health-related quality of life; WHO-5: 5-item World Health Organization Well-Being Index. Adapted from Topp et al [33]. The WHO-5 well-being index scale is not copyrighted and does not require permission to use [38].

Students’ well-being will be assessed longitudinally by the 5-item World Health Organization Well-Being Index (WHO-5) at week 0, week 8, week 10, month 6, and month 12. The WHO-5 well-being index is a short global rating scale that measures subjective well-being for all populations, including adolescents. Positive well-being is another term for mental health (Figure 1) [33]. The key outcome measure includes the positive change in well-being, as estimated by the difference in postintervention (week 8 and week 10) and preintervention (week 0) WHO-5 mean scores by determining the *P* value and effect size using appropriate statistical tests. Although a difference in the mean WHO-5 score of >10 is considered clinically significant [33,34], a meta-analysis of meta-analyses concluded that universal prevention programs of this type in children have effect sizes between 0.07 and 0.16 [35]. Indeed, the difference in the mean WHO-5 score with an antidepressant (desvenlafaxine) vs placebo and wake therapy vs exercise was statistically significant but not clinically significant in patients with depression [36,37].

In children, the hope theory has been introduced on the concept that “goal-directed hopeful thinking” is a requisite for development and survival and is related to experiencing positive emotions [39-41]. Hope within students (≤16 years of age) will also be assessed longitudinally by age-appropriate hope scales at week 0, week 8, week 10, month 6, and month 12. Any improvement in “hope” will be estimated by the difference in postintervention (week 8 and week 10) and preintervention (week 0) mean scores by determining the *P* value and effect size using appropriate statistical tests. Hope theory is related to the theories of learned optimism, self-esteem, and self-efficacy, which are the key goals of the success4life program [42]. Students with “higher hope” have been found to attain higher academic achievement (at all levels of education) and higher graduation rates. They are also better problem-solvers and psychologically adjusted, can handle stress better, are healthier, and have higher self-esteem [43].

**Figure 2:**
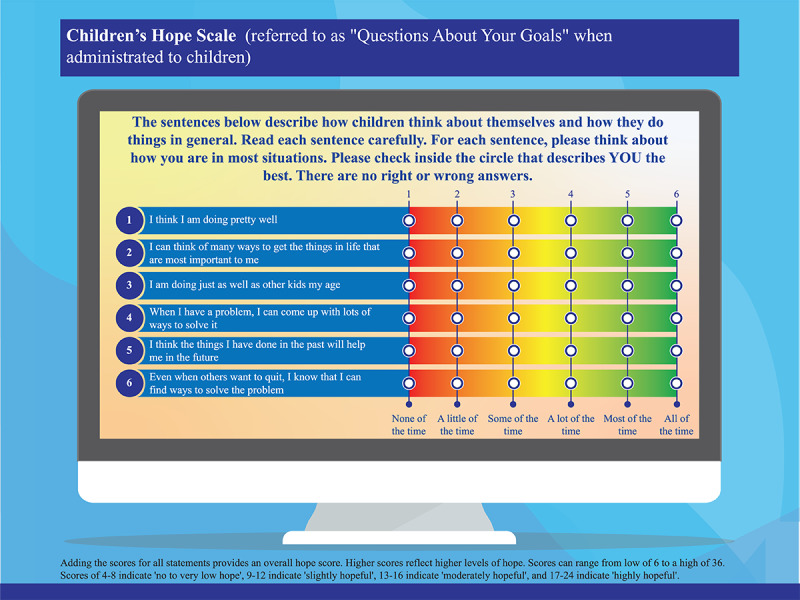
Graphical representation of the Children’s Hope Scale. Adapted from Snyder et al [46]. The Children's Hope Scale is not copyrighted and does not require permission to use [44,45].

**Figure 3:**
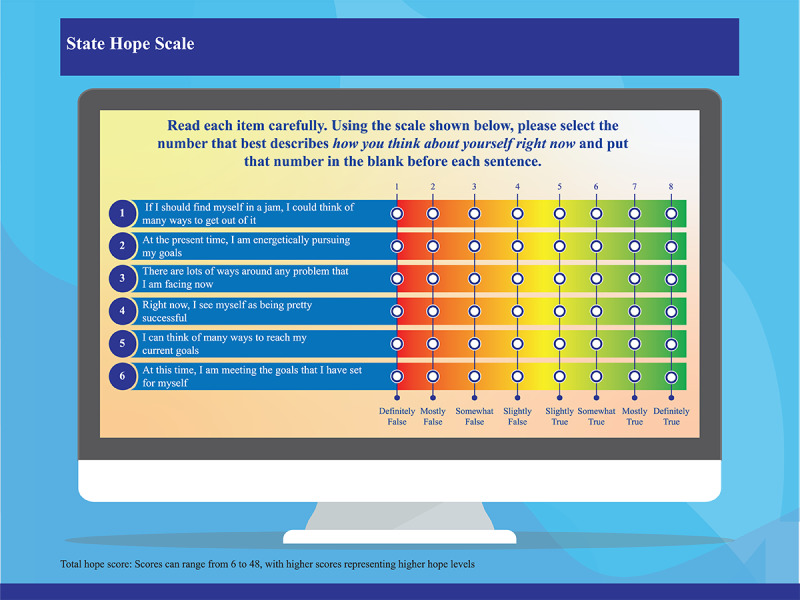
Graphical representation for the State Hope Scale. Adapted from Snyder et al [46,49]. The State Hope Scale is not copyrighted and does not require permission to use [44,50].

Hope in students aged ≤16 years will be assessed using the Children’s Hope Scale (CHS), and the State Hope Scale (SHS) will be used to determine hope for students between 17 and 19 years of age [44]. The CHS measures dispositional hope in children and adopts 6 items on a 6-point Likert scale (Figure 2) [45,46]. The SHS is also a brief 6-item scale. However, responses are rated on an 8-point Likert scale (Figure 3) [47-49].

There are separate WHO-5 and hope surveys at weeks 8 and 10 to analyze the effect of PBL, which will be covered mainly in the last 2 weeks. All the scales (WHO-5, CHS, and SHS) have been validated, are not copyrighted, and as such do not require permission to use [38,45,50]. Figures 1, 2, and 3 provide a graphical representation of the WHO-5, SHS, and CHS, respectively

At the end of each workshop in weeks 1-8, the students will also rate the effectiveness of each training session (as the usefulness of training in day-to-day life and ability of the movement in achieving its objectives), report a net promoter score as a metric to study learner satisfaction, and evaluate the performance of the trainer using a brief anonymous closed-ended postworkshop web-based questionnaire. Descriptive statistics will compare the students’ aggregate responses for each week.

Surveys will be conducted in accordance with the global standards of good clinical practice (as defined by the International Conference on Harmonization E6 Guidelines for Good Clinical Practice) [51], the latest version of the Declaration of Helsinki, and the national regulations [52].

### Ethics Considerations

At week 0, interested participants’ legal guardians will complete the “enrolment agreement form” as part of the onboarding process for parents and guardians. Informed consent will be obtained from the legal guardians of students. The program’s effectiveness will be assessed by a series of web-based questionnaires conducted on recruited students whose parents or guardians provided informed consent for the study. All the research questionnaires directed to the students are entirely anonymous, voluntary, and close-ended.

The research methodology is exempt from the requirement of ethics committee approval according to the Office for Human Research Protections, US Department of Health and Human Services, as the survey questionnaires will be completely deidentified [53,54]. Any suspected cases of justifiable concerns related to physical or mental health will be brought to the attention of relevant responsible party (eg, school counselors, parents, or legal guardians).

## Results

The study has 2 phases. A pilot phase (phase 1) will involve the recruitment of approximately 100 adolescent students between the ages of 13 and 19 years from the United States. Coaching will be preferably digitally and mainly conducted by the creators of the modules (2 physicians and a psychology educator with a doctorate). Phase 2 will involve training the trainers program. The “new coaches” could be teachers and peer mentors (with nonscientific backgrounds) or psychology, medical, or science graduates and postgraduate students who will contribute to making a larger social impact once the program is fit to scale. The target number of participants in phase 2 will be between 500 and 1000. Results from phase 1 will serve as an external control to test the effectiveness of the scalability platforms. The sample size predictions for phases 1 and 2 are based on a similar PPI, the Hummingbird Project from the United Kingdom, which led to improvements in adolescents’ well-being, also measured using the WHO-5 [18]. We anticipate to start student recruitment by 2022 through partnering with schools, family, and youth organizations and aim to obtain the results of phase 1 pilot testing by 2023.

Overall, we expect to see positive effects on students’ well-being and to enhance competency for essential life skills, including coping with adversities through learned optimism, decision-making, problem-solving, resisting peer and social media influences, and self-control through our success4life program.

## Discussion

The scientific breakthroughs through effective vaccinations and medications have helped prevent severe COVID-19 infection and deaths [55,56]; however, much of the work remains unfinished until we effectively tackle the rising mental disorders in the youth [57]. The challenge is to continue to direct innovation and science to effectively “flatten the curve” of increasingly alarming psychological distress in the youth, especially adolescents [15,58].

In this paper, we describe a holistic program developed by our team aimed to tackle the key challenges faced by adolescents in the modern age with technology-enhanced, real-time, hands-on experience on positive psychology. Our program uses IBL and PBL techniques to provide teenagers and emerging adults with methods for adaptive coping for relieving stress, self-compassion, goal attainment, learned optimism and resilience, and self-protection. In addition, we include modules on the awareness of the dangers of substance use and vigilance on social media. To our knowledge, this is the first time such a program has been developed, and we propose here a method to test its effectiveness and scalability.

Real-world data (RWD) consist of information collected during routine clinical, counseling, or coaching practice [59-61]. Real-world evidence (RWE) originates from studies based on valid RWD collected outside traditional controlled clinical trial programs. RWD, however, involves inherent biases and must be fit for the purpose of generating RWE. Challenges of RWD include data quality, reproducibility, replicability, and accuracy, which may affect validity [59].

The research methodology for the generation of RWD includes implementation science, qualitative research, translational research, participatory research, surveys, precision medicine, and mixed methods research [60]. Deidentified anonymous data enable researchers to study the effectiveness of mental health interventions, treatment, and services by observing outcomes recorded within routine sessions [61].

Two serious concerns with RWD include reproducibility and replicability [62]. Ideally, a study testing an intervention should be reproducible through not only direct replication (adopting the same methodology) but also conceptual replication (adopting a different methodology) [63]. For a universal PPI to have a viable future and make a public impact, it needs to be scaled up and delivered by multiple trainers on multiple sites [18]. Our main research will check the effectiveness of the same intervention (success4life program) upon scaling through the *train the trainer* program for conceptual replication of the intervention. Minor amendments in the intervention to fix any inefficiencies or flaws, as evidenced by pilot testing, are permitted and will be documented.

Students are generally reluctant to answer IBL questions about their own experiences or opinions, not only owing to fears about revealing personal information to peers but also because they are often afraid that they may give “wrong” answers [64-66]. To overcome this hesitancy, all IBL journal completions for our intervention are real-time, digital, technology-based submissions through an LMS that protects the student’s anonymity and privacy to peers. A clear statement that there are no right or wrong answers will be implemented in all journals during each workshop. The PPIs designed to improve well-being are often compared to waitlist controls, which leaves ambiguity and unmeasured confounders related to their effectiveness [67]. Hence, we will not have any separate control group and will only study the change from baseline.

Randomized controlled trials (RCTs) with homogeneous study populations and double-blind study designs with statistical sample size and study calculations before the start of the study are the gold standard for determining the efficacy of interventions [68]. Our study lacks an RCT study design and a statistical sample size calculation, which could introduce bias. We decided to adopt an RWD design because RCTs on universal social and emotional learning programs have shown controversial findings [69], as often digital interventions show meaningful improvements only when tested under strictly controlled research settings [70]. Furthermore, although RCTs carry a high internal validity, the external validity or generalizability of the results to a wider population is always a concern with RCTs owing to strict inclusion and exclusion criteria [68,71].

Nonetheless, real-world studies on heterogeneous populations without randomization are subject to bias and confounders [71]. The scientific best practices roadmap we adopted for scientific strategies needed to draw valid causal conclusions on the effectiveness of the success4life program includes the adoption of robust and validated study end points and publishing of the study protocol to enhance study transparency. We also use neutral instead of negatively or positively phrased items and measures to avoid central tendency bias (in Likert scales) for the weekly workshop feedback questionnaires, using closed questions in the survey. We decided to make the response to all questions mandatory for submission of each feedback questionnaire to avoid any issues of missing values in data sets and to keep the surveys fully anonymous yet voluntary to prevent nonresponse bias [59,63,72,73].

Pessimistic explanatory style (self-talk and the way people explain to themselves why they experience bad or good events) leads to cognitive vulnerability to depression, poor health, and early mortality [74,75]. Many factors—for example, the explanatory style of parents (especially that of mothers) for their and their children’s events along with childhood trauma, chronic adversity, and abuse—influence the development of a pessimistic explanatory style as a trait [74-76]. Interventions aimed at changing a pessimistic explanatory style and outlook enhance mental and physical well-being and lower the likelihood of future illnesses [76,77]. This is also relevant for breaking the cycles of intergenerational trauma and ever-increasing psychological distress in the youth by promoting protective processes that not only buffer against life’s uncertainties but also intergenerational risk and provide immunization against mental health disorders [78,79].

## Appendix

Multimedia Appendix 1 Broad overview of each workshop's specific details, including the theme, objectives, and specific IBL and PBL exercises. IBL: inquiry-based learning. PBL: project-based learning.

## Abbreviations

ABCDE: Adversity-Beliefs-Consequences-Disputation-Energization
CHS: Children’s Hope Scale
IBL: inquiry-based learning
LMS: learning management system
MSUD: mental and substance use disorder
PBL: project-based learning
PPI: positive psychology intervention
RCT: randomized controlled trial
RWD: real-world data
RWE: real-world evidence
SHS: State Hope Scale
WHO-5: 5-item World Health Organization Well-Being Index

## Data Availability

Multimedia Appendix 1 contains a PDF document entailing an infographic-based broad overview of the theme, module-specific complete details, and research phases of the study.
