# Supplementary material for: Success4life Youth Empowerment for Promoting Well-being and Boosting Mental Health: Protocol for an Experimental Study
Source: JMIR Res Protoc. 2022 Sep 14;11(9):e38463. doi: 10.2196/38463 (PMC9520395; doi:10.2196/38463)
Supplement: Multimedia Appendix 1 [file resprot_v11i9e38463_app1.pdf]

Urgency for a scalable intervention for alarming psychological distress

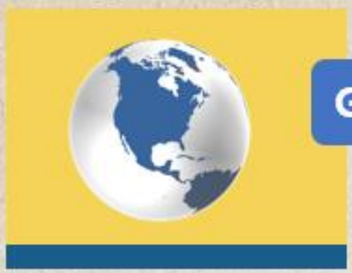

Globally, suicide is listed as the second leading cause of mortality in adolescents and young adults.[1]

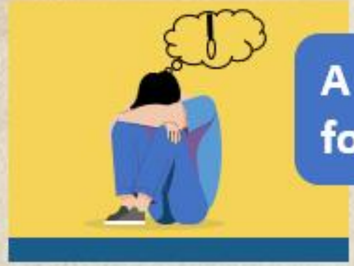

A cross-national pooled estimate of suicide mortality rate across all ages, sexes, and countries was found to be 3.77/100,000 people in 2019.*[2]

*however, underestimation and underreporting of youth suicide impacts the accuracy of suicide epidemiology

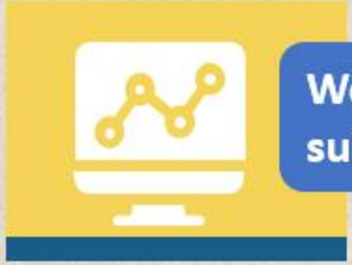

Worldwide data indicates a further increase in mental health-related emergency visits reported suicidal ideation and behavior in the youth after the COVID-19 pandemic.[1,3-6]

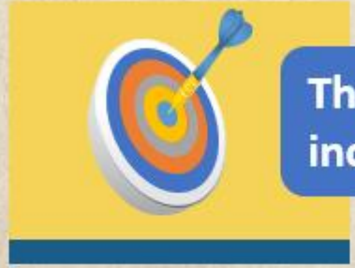

The challenge now is to continue to direct innovation and science to effectively '<u>flatten the curve</u>' of increasingly alarming psychological distress in the youth, especially adolescents.[7,8]

1. Asarnow JR, et al. *Journal of child psychology and psychiatry, and allied disciplines*. 2021;62(8):919-21.
2. Glenn CR, et al. *Journal of child psychology and psychiatry, and allied disciplines*. 2020;61(3):294-308.
3. Manzar MD, et al. *J Child Adolesc Psychiatr Nurs*. 2021;34(2):139-46.
4. Gracia R, et al. *J Affect Disord*. 2021;292:139-41.
5. Schwartz-Mette RA, et al. *J Clin Child Adolesc Psychol*. 2022:1-14.
6. Lantos JD, et al. *Pediatrics*. 2022;149(2).
7. Ridout KK, et al. *JAMA psychiatry*. 2021;78(12):1319-28..
8. Alkhayyat A, et al. *Disaster medicine and public health preparedness*. 2020;14(4):e44-e5.

Success4life: a holistic approach to improve coping skills in adolescents

Technology-enhanced hybrid interventions continue to hold promise for increasing engagement in and enhancing outcomes of evidence-based approaches

A hybrid PPI, 'success4life youth empowerment', that adopts unique benefits from both real-time synchronous and asynchronous learning and overcomes the unique limitations of each mode of coaching.

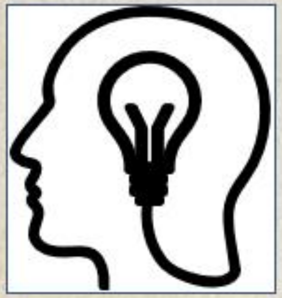

This is a holistic program in the spectrum of coverage of elements of positive psychology and targets unique risk factors related to high-risk behavior, commonly encountered in this age group.

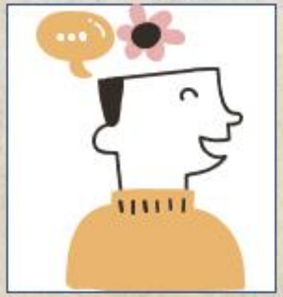

We aim to check the effectiveness of this PPI in improving well-being and levels of hope in the youth, as well as checking satisfaction for each PPI workshop by the learners.

PPI, Positive psychology interventions

Program architecture

We have earlier published an evidence-based course curriculum for the universal prevention of MSUDs in the youth.[1]

We have now built the modules, systems, and processes for effective delivery of the success4life program for adolescents and pipeline for emerging adults.

The entire curriculum is designed to provide coaching on adaptive coping for relieving stress, self-compassion, goal attainment, learned optimism and resilience, self-protection.

Coaching methodology includes hands-on experience on positive psychology with inquiry and project-based, student-directed learning through reflection within real-world experiences and problem-solving.*

The modules are created using evidence-based, scientific information published in peer-reviewed medical and scientific journals and esteemed books.

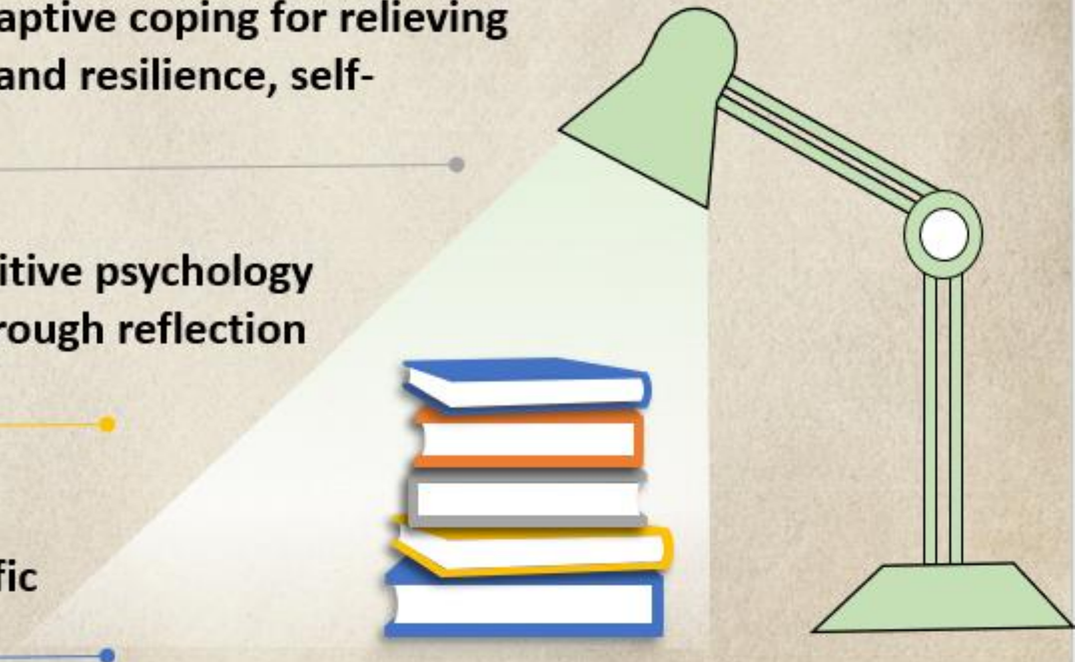

MSUDs, mental and substance use disorders .

1. Setia S, et al. *Alternative and Complementary Therapies.* 2021;27(2):68-78.

**The content incorporated in each module and learners' journal template is protected by intellectual property, granted to Transforming Life LLC, United States (United States copyright office, registration number: TXu 2-327-132; approval date: July 27, 2022).*

Our Learning Model

Transforming life

Learning model comprises of blended digital learning

Synchronous live real-time training

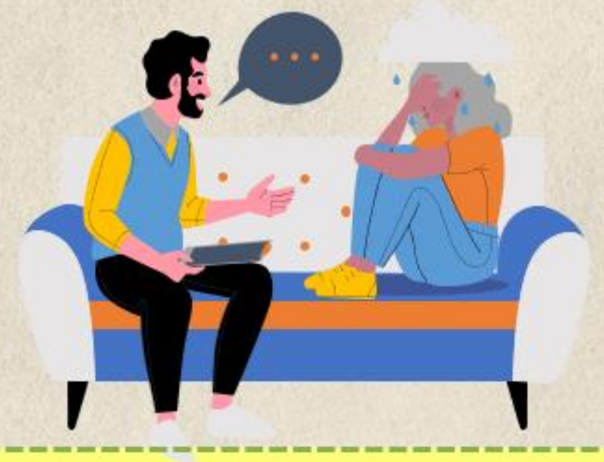

- Allows sufficient contact with the instructors for effective interactive coaching and learning.
- Total duration for each weekly session of live training is between 60-90 minutes.
- Can be online/offline face to face meeting.
- A structured approach for simultaneous project completion during each week's training is followed.
- Brief 5 min. break to complete IBL exercises.

Asynchronous e-learning via an LMS

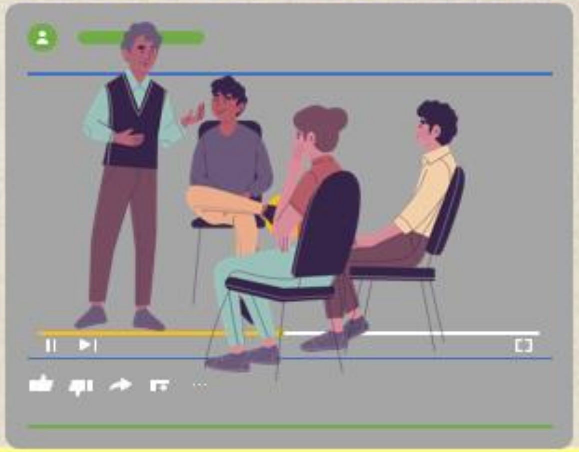

- Hosts video modules for asynchronous refresher education.
- Tracks data related to learner's assignment submission, evaluation as well as workshop completion certification.
- Separate PBL, as relevant for some modules, is adopted to enable learners' practical investigations, independence, and reflection within real-world practices.

LMS, Learning Management System; IBL, inquiry-based learning; PBL, project-based learning.

Modules of the course

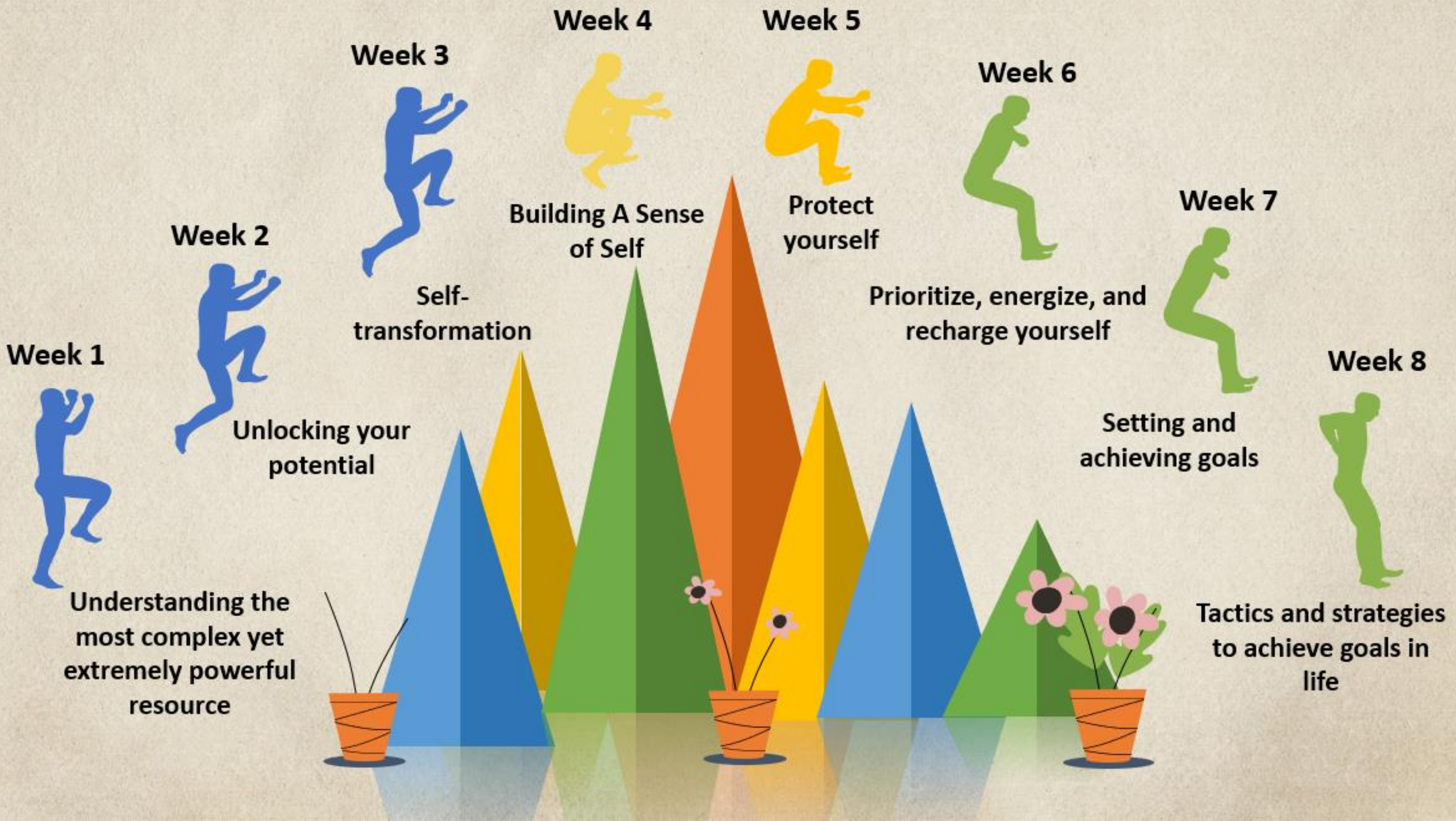

Week 1 Workshop

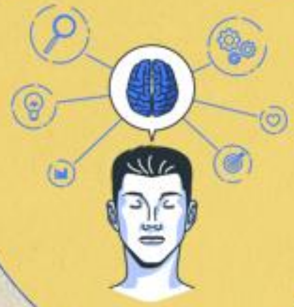

UNDERSTANDING THE MOST COMPLEX YET EXTREMELY POWERFUL RESOURCE

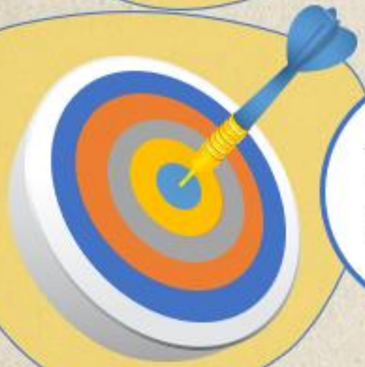

To give students an awareness and appreciation of the innate potential in each of us, which can be harnessed by learning to control and shape our mind and thought process.

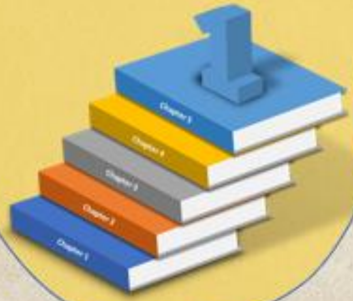

There are three lessons included:

(i) Understanding our mind;
(ii) Mind-body problem; and
(iii) The power of our mind

Week 1 Workshop: IBL topics

Transforming life

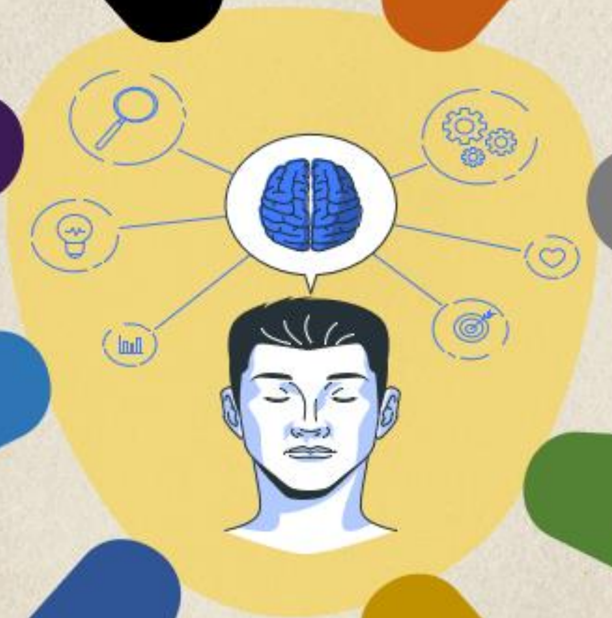

1. How do they see their mind and consciousness working together?
2. How often are they consciously aware of their emotions?
3. How do they express, release and manage your core emotions?
4. How do they experience mind-body relationship?
5. Recollecting a memory when they were able to use positive emotions to create physical well-being or healing for yourself
6. How do they experience and handle the state of 'Monkey Mind?
7. Personal rating for each of the elements of emotional intelligence (EQ)
8. Which elements need more attention for improving their overall EQ'

IBL, inquiry-based learning.

Transforming life

Week 2 Workshop

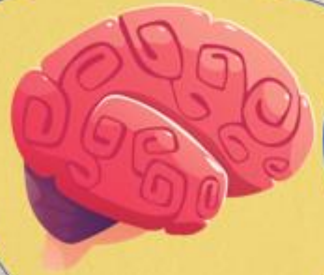

UNLOCKING YOUR POTENTIAL

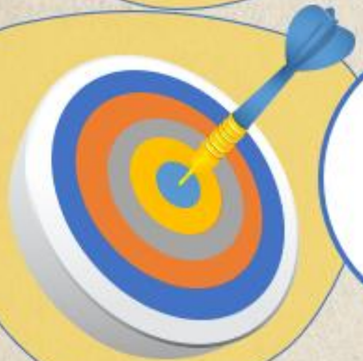

- **To provide an understanding related to automatic, unconscious thought processes that are generated with no or very little awareness and consciousness.**
- **The students also learn how to foster a sound connection between the conscious and unconscious minds for success and happiness in life.**

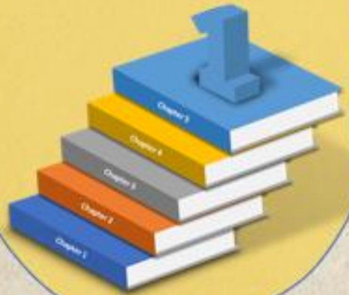

There are three lessons included:

(i) Understanding our unconscious mind;

(ii) Reprogramming our unconscious mind; and

(iii) How to apply knowledge in real-life situations

Week 2 Workshop: IBL topics

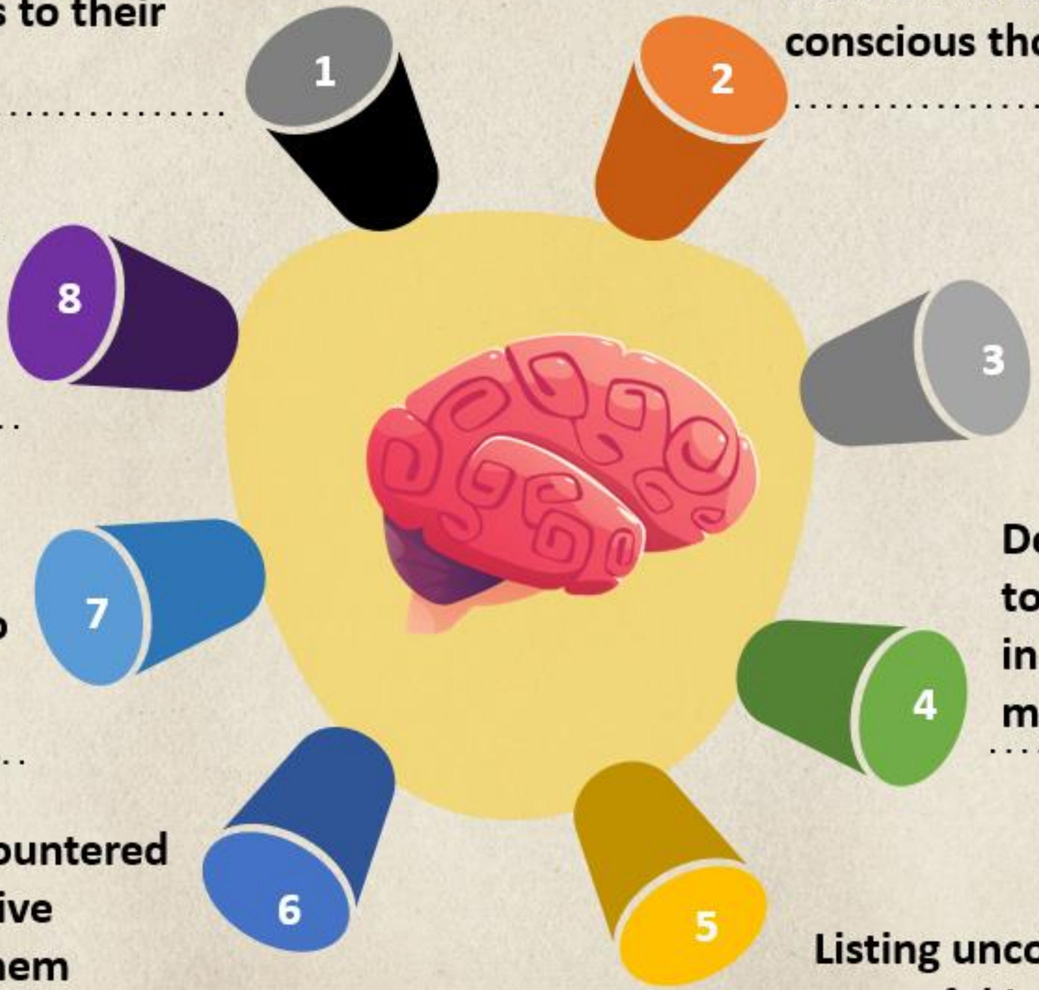

1. Creating a mind map of their unconscious mind and how it connects to their conscious thoughts
2. How is this connected to their conscious thoughts?
3. Create a story/ doodling what represents their unconscious mind as their warehouse of hidden beliefs'
4. Describing a time that they chose to challenge an unconscious belief in order to direct themselves in a more positive direction'
5. Listing unconscious influences that are powerful in their life (social media, etc.)
6.
   - Listing some frequently encountered negative thoughts and positive alternatives to counteract them
   - Positive personal affirmations
7. Creating positive energy plan to keep themselves motivated
8. A project assigned on creating a 'magic wand' as a representation of self-encouraging beliefs

IBL, inquiry-based learning.

Week 3 Workshop

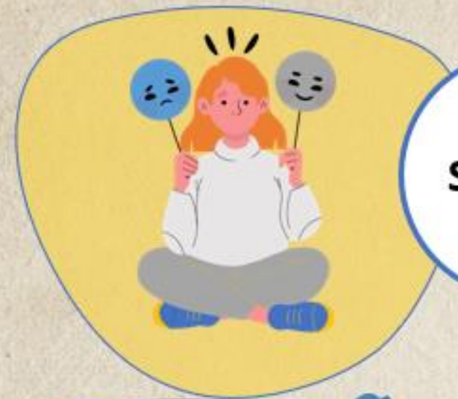

SELF-TRANSFORMATION

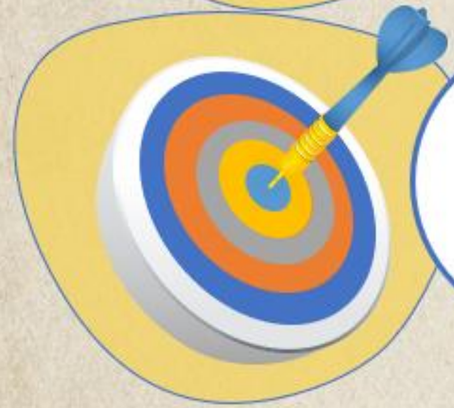

The students learn why and how to expand our consciousness to achieve self-transformation with a deeper fulfillment and purpose in life.

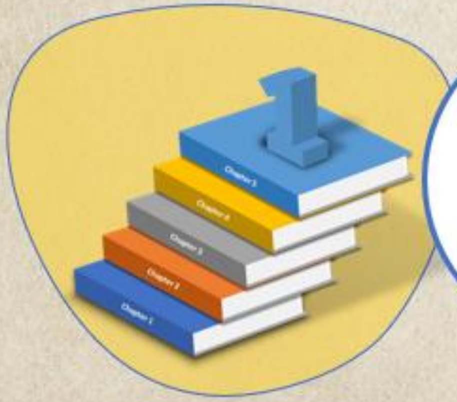

There are three lessons included:

(i) What is self-transformation;

(ii) Relationship between self-transformation and happiness, abundance, and success; and

(iii) Transforming the old self into a new empowered self

Week 3 Workshop: Steps and IBL topics

Before learning the meaning of self-transcendence, students reflect on exploring meanings of passion and purpose.

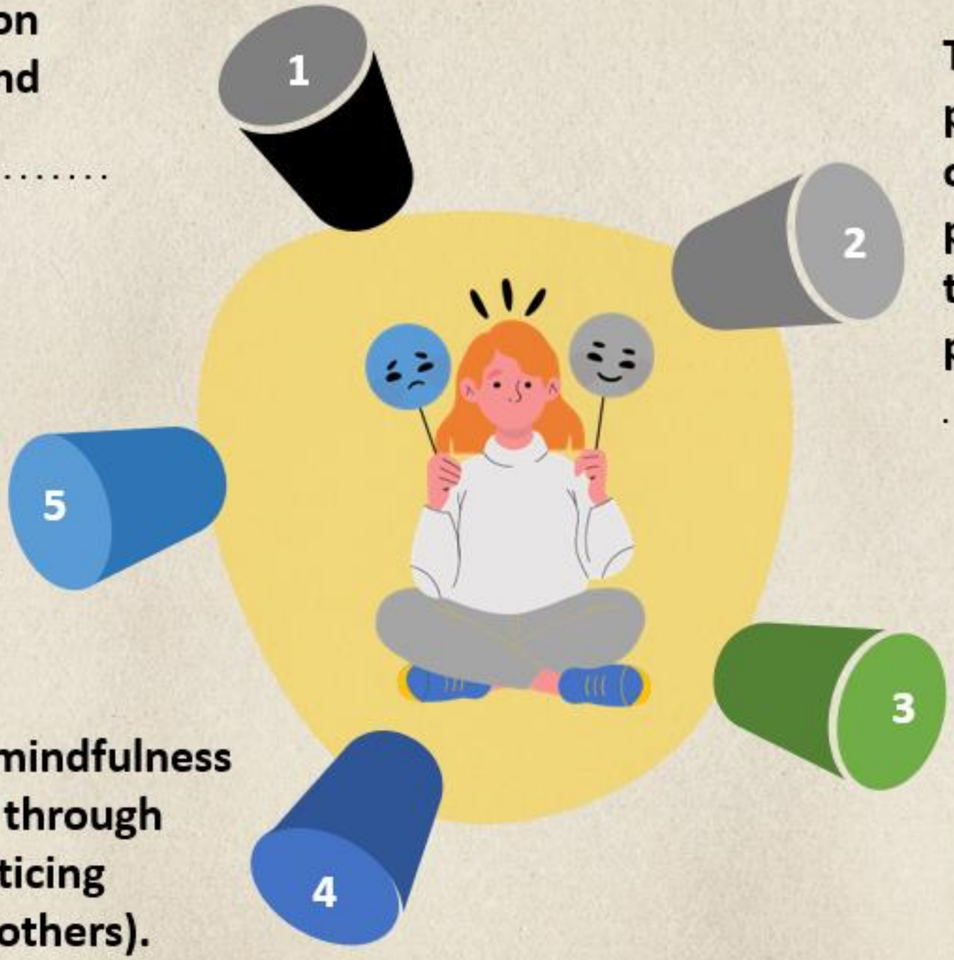

They try to define passion and purpose in their own words, reflect on what they are especially passionate about and note down their current belief about their own purpose in life.

The students then create their own “self-transformation” or “hero’s journey” in their journal, listing their greatest goals in life, their core values and emotions that they are seeking to cultivate in life.

They learn the fundamentals of mindfulness and recognize distorted thinking through mindfulness, gratitude, and practicing forgiveness (for themselves and others).

For the project, the students are assigned to spend 10 minutes a day engaging in these activities.

IBL, inquiry-based learning.

Week 4 Workshop

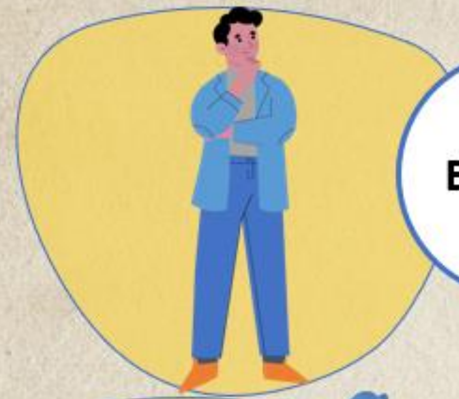

BUILDING A SENSE OF SELF

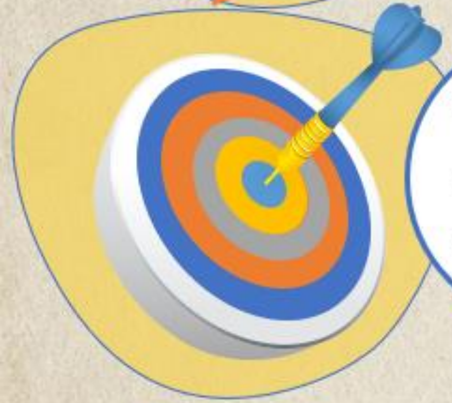

To give students an awareness and understanding of how building self-esteem through self-compassion relates to positive outcomes in life and learn strategies to improve our self-esteem and self-confidence.

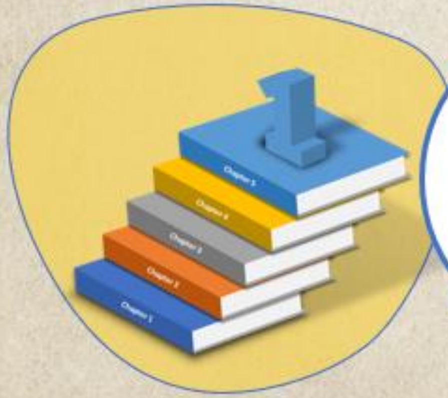

There are three lessons included:

(i) Understanding self-image, self-esteem, and self-compassion;

(ii) Building self-esteem and self-confidence through self-compassion; and

(iii) Practicing self-love and self-care

Week 4 Workshop: IBL topics

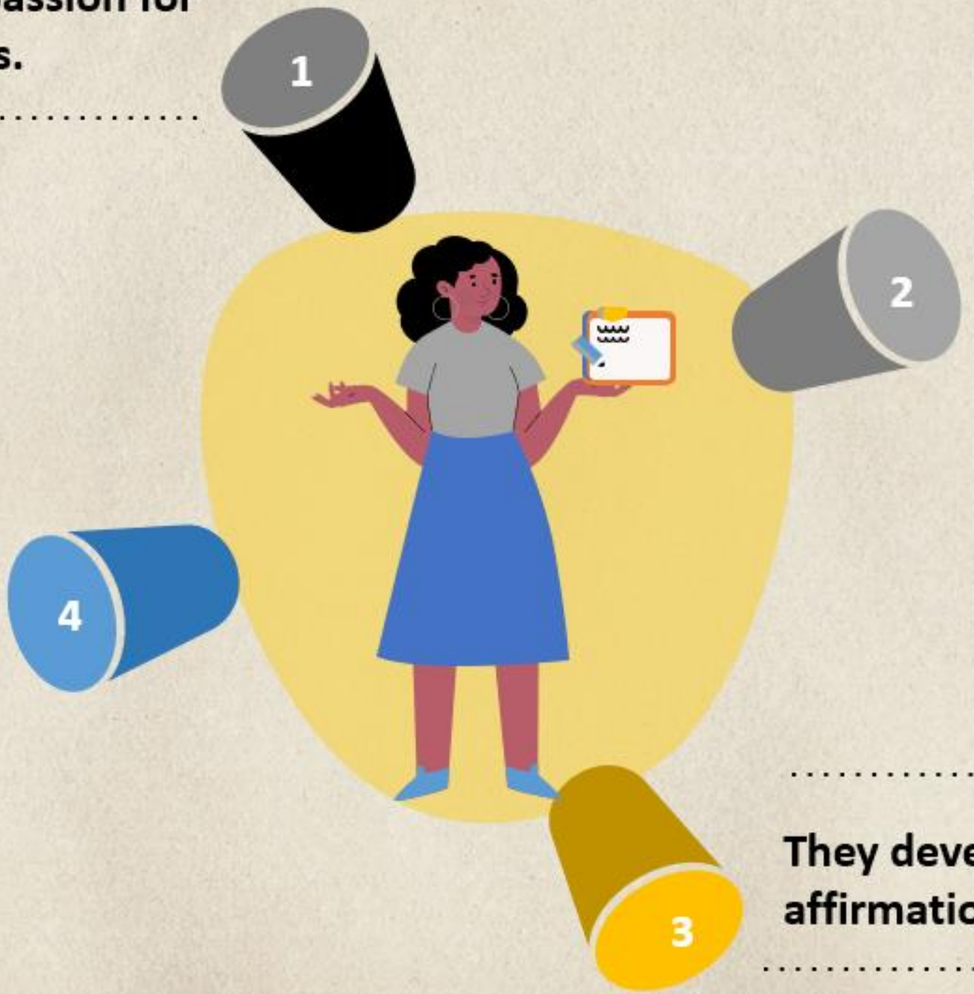

1. Students express self-compassion for themselves in their journals.
2. Write/ illustrate ways to bring more care, kindness, and support for themselves into their daily life.
3. They develop their affirmations.
4. Create some goals for themselves to practice self-love and self-care.

IBL, inquiry-based learning.

Week 5 Workshop

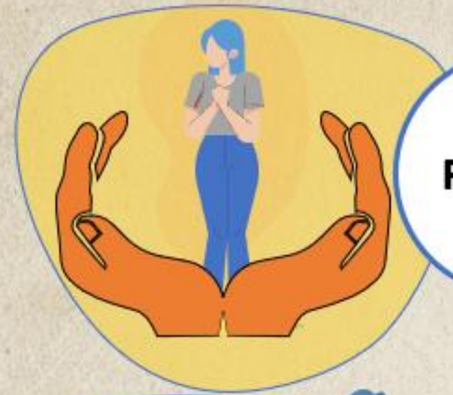

PROTECT YOURSELF

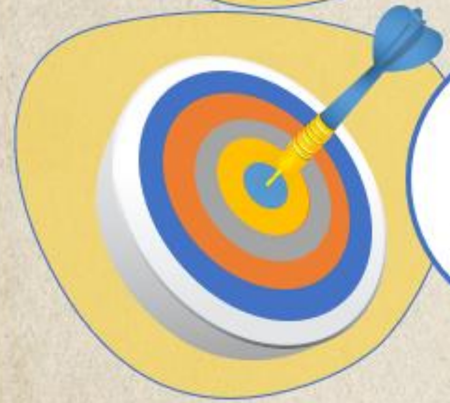

- **To give students an awareness and understanding of how our connections through friends, peers as well as social media influence our thoughts, choices, and decisions in every way.**
- **The impact may be positive or negative, but often we are not conscious of this dominance.**

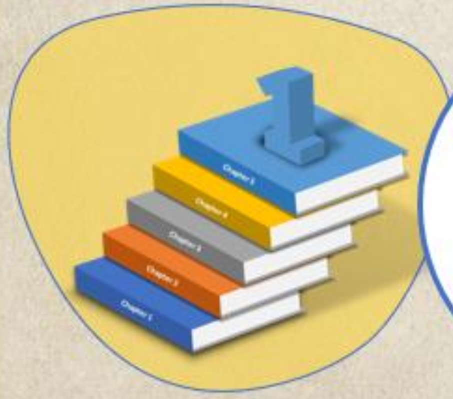

There are four lessons included:

(i) Conscious connections and due diligence;
(ii) Stay safe - substance abuse awareness;
(iii) It is okay to say no; and
(iv) Staying vigilant on social media

Week 5 Workshop: Steps and IBL topics

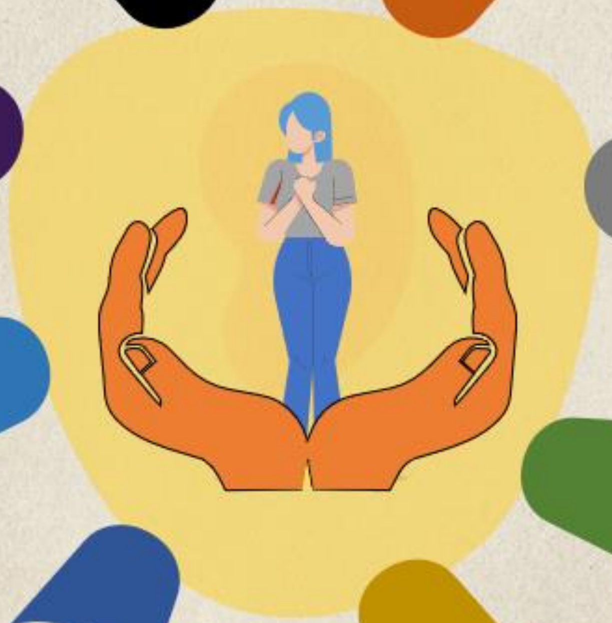

1. Students learn how to exercise independence in their decisions without pressure from peers and resist negative influences.
2. Students are coached through common real-life case studies on how to make independent decisions without the pressure to please their peers.
3. The importance of states of hot and cold cognition in teenage years.
4. Why and how to keep themselves away from possible addictions?
5. To develop a good 'sleep plan'.
6. Best practices with use of mobile and electronic devices (including discussion on common issues related to 'sexting' and its moral and legal complications).
7. One of the activities is for each student to develop a superhero battle plan to tackle social media hazards.
8. Students reflect on what they can do to make their life more meaningful for themselves and others.

IBL, inquiry-based learning.

Week 6 Workshop

Transforming life

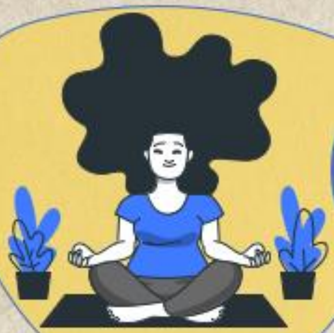

PRIORITIZE, ENERGIZE, AND RECHARGE YOURSELF

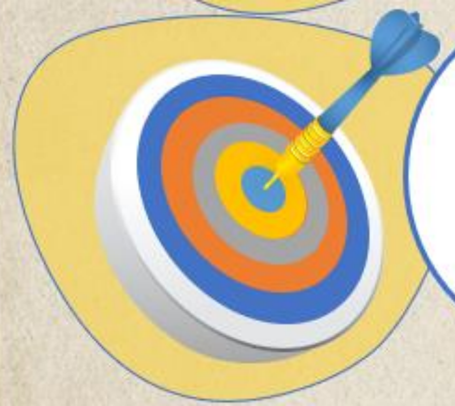

- **The students understand what it is like to practice reflection on their thinking with no or as little judgment as possible, allowing them to capture each moment with an awareness of change.**
- **They also learn to detach from non-beneficial thoughts such as anger and judgment and let go of unwanted emotions.**

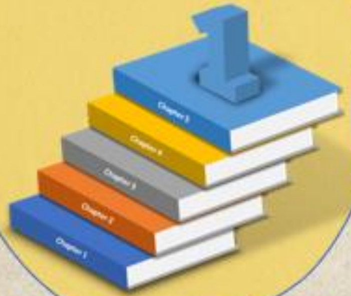

There are three lessons included:

(i) Building self-esteem, optimism, and resilience with mindfulness practices;
(ii) Practicing the shield of mindfulness; and
(iii) Meditation exercises

Week 6 Workshop: Steps and IBL topics

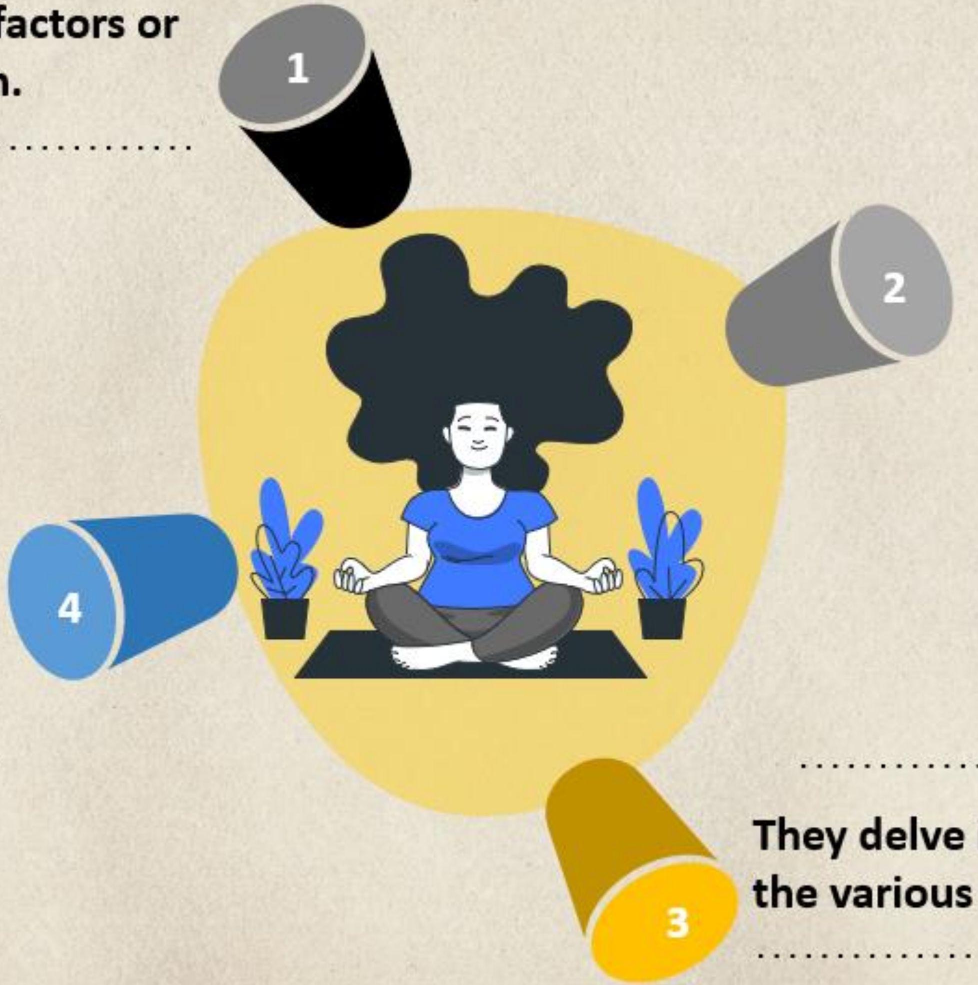

1. The students think and identify factors or triggers that bring stress to them.
2. The students analyze as to what they can do to better deal with stressful situations in the future.
3. They delve into meditation exercises and learn the various formal and informal meditations.
4. They explore and observe their recent thinking pattern like a scientist and explore their own preferred style of practicing mindfulness.

IBL, inquiry-based learning.

Week 7 Workshop

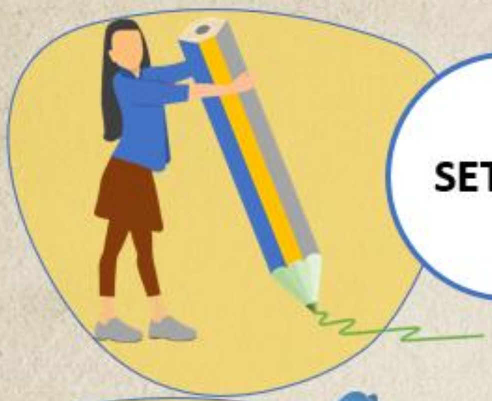

SETTING AND ACHIEVING GOALS

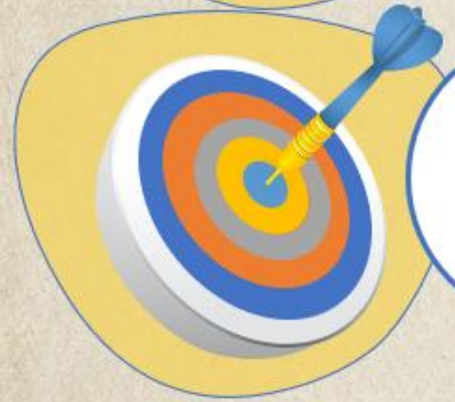

The students understand the key elements related to short- and long-term goals and build on setting personal goals for both short-term and long-term.

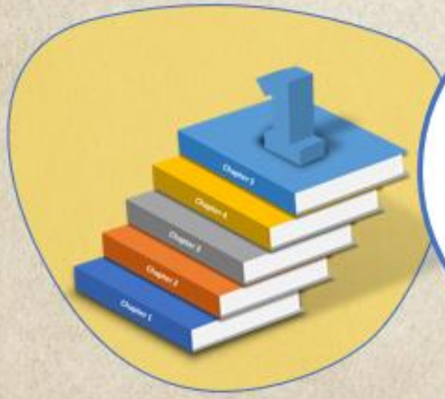

There are four lessons included:

(i) Discover your passion and purpose;
(ii) Long-term goals;
(iii) Short-term goals; and
(iv) What stops us from achieving our goals?

Week 7 Workshop: Steps and IBL topics

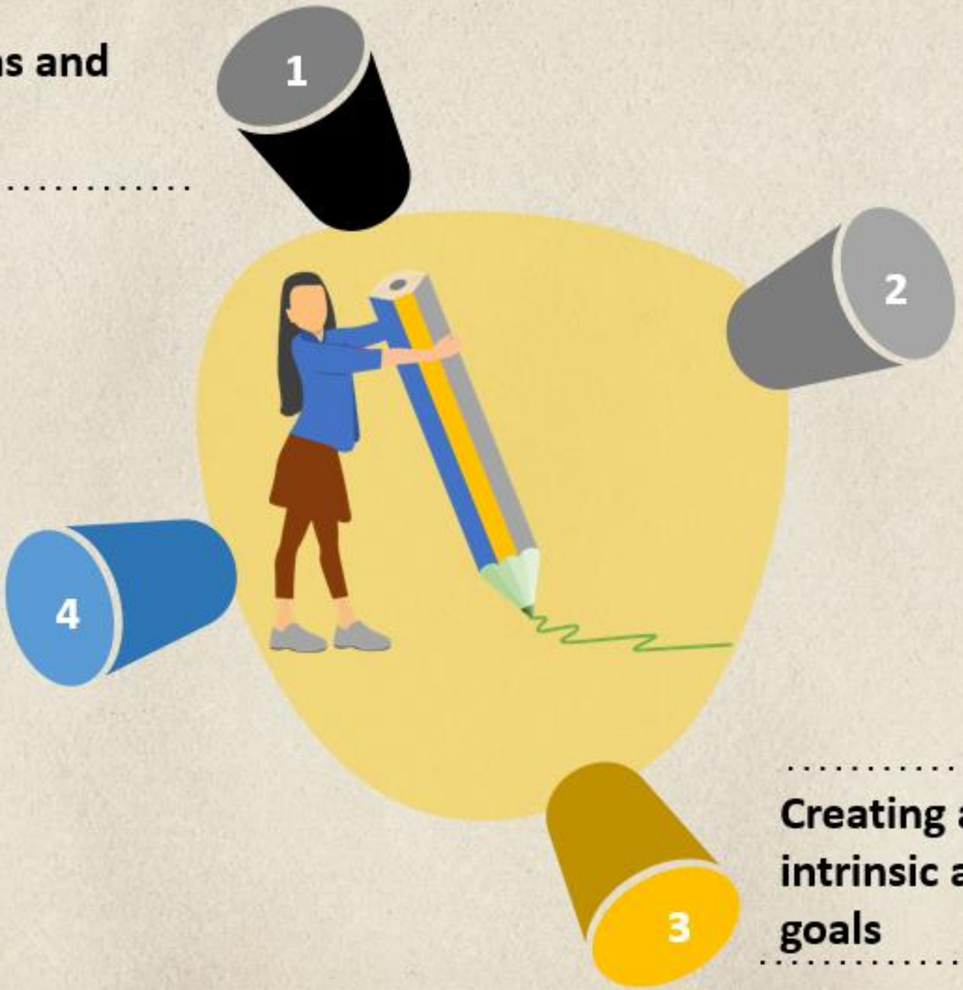

1. **Exploring their own passions and life purpose**
2. **Listing the top 3-5 things they are passionate about**
3. **Creating a visual representation of intrinsic and extrinsic long-term goals**
4. **Creating at least three SMART short-term goals, etc.**

IBL, inquiry-based learning; SMART, Specific- Measurable-Attainable-Realistic-Time-bound.

Week 8 Workshop

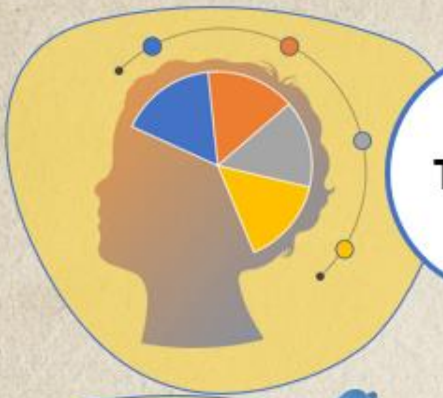

TACTICS AND STRATEGIES TO ACHIEVE GOALS IN LIFE

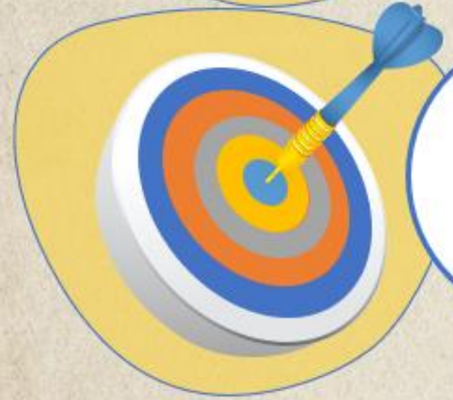

To explain optimism and introduce students to a model to cope with life adversities, teach how to overcome negative thoughts as well as how to turn adversities into opportunities with accurate choices and decisions

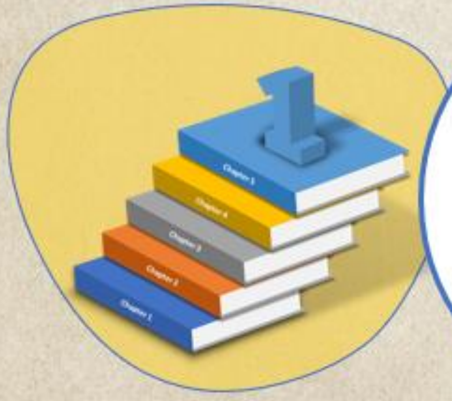

There are four lessons included:

(i) Definition and meanings of 'optimism' and 'resilience';

(ii) ABCDE (Adversity-Beliefs-Consequences-Disputation-Energization) model to cope with life adversities;

(iii) Transforming your self-talk; and

(iv) How to turn adversities into opportunities with accurate choices and decisions?

Transforming life

Week 8 Workshop: Steps, IBL topics and PBL

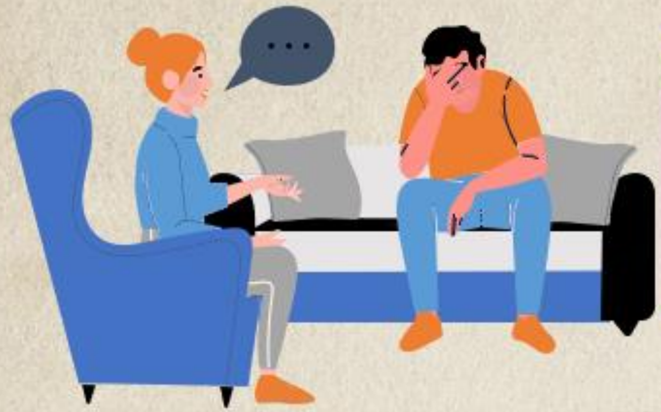

- Students are provided with an understanding of 'explanatory styles' in simple terms and differences between negative and positive explanatory styles',
- Benefits of optimism as a trait and concepts related to learned optimism, and differences between optimism and unrealistic optimism.

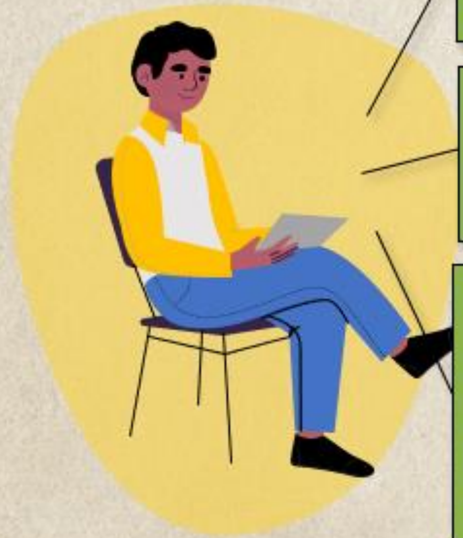

The students describe the terms 'optimism' and 'resilience' using their own words.

Explain life situations from an optimist and a pessimist viewpoint.

Case studies, discussion related to automatic negative thoughts, positive empowering thoughts, and growth mindset and IBL related to the case studies.

Lastly, the students are assigned a project (Mind Power Tool) for the week to create a similar fictitious character using their journal, an eBook, or a video narrating the concept and the story

The story is based on how the character overcomes automatic negative thoughts and overcomes obstacles representing the scientific basis for the law of attraction in life

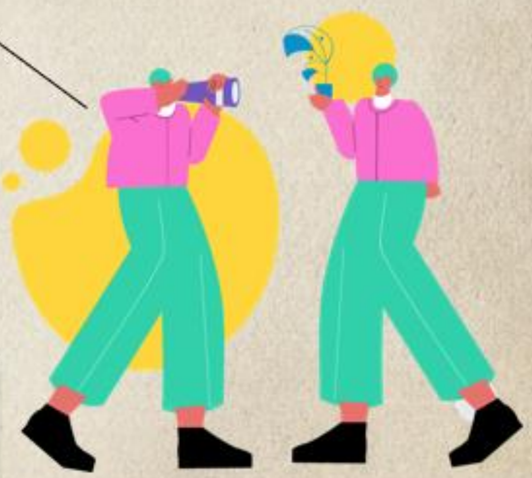

IBL, inquiry-based learning; PBL, project-based learning.

Study Phases

Phase 1

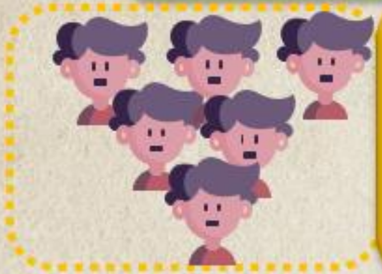

Recruitment of about 100 adolescent students between the age of 13-19 years from the United States.

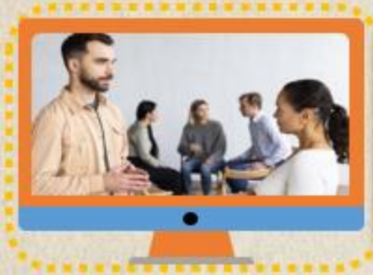

Coaching will be preferably online and mainly conducted by the creators of the modules (two physicians and a psychology educator with a doctorate).

Phase 2

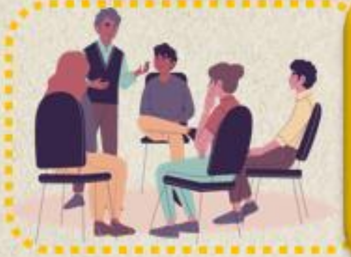

It will involve train the trainers' program, and the 'new coaches' could be teachers and peer mentors who will contribute to making larger social impact once the program is fit to scale.

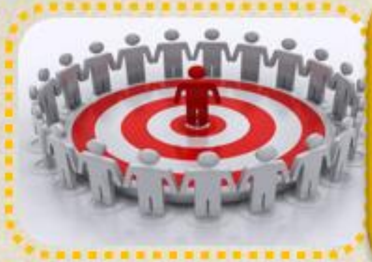

The target number of participants in phase 2 will be between 500-1000.

- **Results from phase 1 will serve as an external control to test the effectiveness of the scalability platforms.**
- **The sample size predictions for both phases 1 and 2 are based on a similar PPI, Hummingbird Project from the United Kingdom[1], that led to improvements in adolescents' well-being, also measured by the WHO-5 index.**

PPI, Positive psychology interventions

1. Platt IA, Kannangara C, Tytherleigh M, Carson J. The Hummingbird Project: A Positive Psychology Intervention for Secondary School Students. Front Psychol. 2020 Aug 7;11:2012.
